# Anlotinib alone or in combination with bevacizumab in the treatment of recurrent high-grade glioma: a prospective single-arm, open-label phase II trial

**DOI:** 10.1186/s12885-023-11776-4

**Published:** 2024-01-02

**Authors:** Shuangshuang Zhao, Minmin Zhang, Qing Zhang, Jingjun Wu, Hui Dai

**Affiliations:** Department of Radiation Oncology, Hangzhou Cancer Hospital, No.34, Yanguan Lane, Shangcheng District, Hangzhou, Zhejiang 310002 China

**Keywords:** Anlotinib, Bevacizumab, Multi-target tyrosine kinase inhibitor, Recurrent high-grade glioma

## Abstract

**Background:**

Anlotinib is a multi-target tyrosine kinase inhibitor (TKI) targeting the vascular endothelial growth factor receptor (VEGFR), platelet-derived growth factor receptor (PDGFR), fibroblast growth factor receptor (FGFR), and c-Kit. This phase II study aimed to assess the efficacy and safety of anlotinib, either alone or in combination with bevacizumab (Bev) for recurrent high-grade glioma (rHGG) (NCT04822805, 30/03/2021).

**Methods:**

Eligible patients had a histological diagnosis of rHGG with first or subsequent recurrences. All patients received oral anlotinib 12 mg or 10 mg on days 1–14 (repeated every 21 days). In cases where brain magnetic resonance imaging examination revealed an increase in peritumoral edema without worsening of symptoms, patients received a temporary treatment of intravenous bevacizumab 10 mg/kg to alleviate edema. The primary endpoint was the median progression-free survival (mPFS), and the secondary endpoints included median overall survival (mOS), objective response rate (ORR), disease control rate (DCR), and safety.

**Results:**

Twenty-five patients with rHGG were included in the efficacy and safety assessments. Eighteen patients received anlotinib alone, and seven patients received anlotinib in combination with Bev. For all patients, the mPFS and mOS were 5.0 months and 13.6 months, respectively. The ORR was 32%, and the DCR was 96%. It is noteworthy that the survival and response data of recurrent glioblastoma (rGBM) exhibit similarities to those of rHGG. For rGBM patients, there were no significant differences in mPFS, mOS, ORR, or DCR between the anlotinib alone and anlotinib + Bev groups. However, the incidence of treatment-related adverse events of any grade was higher in the anlotinib + Bev group compared to the anlotinib alone group (100% vs. 78%, *p* = 0.041).

**Conclusions:**

Both anlotinib alone and its combination with Bev demonstrated good efficacy and safety in the treatment of rHGG.

## Background

Glioma has an annual incidence of 3 ~ 6.4/100,000, representing approximately 78.3% of all malignant tumors in the central nervous system (CNS) [1]. According to the World Health Organization (WHO) classification, glioma is divided into four grades, with grades 1 and 2 being referred to as low-grade glioma (LGG), and grades 3 and 4 as high-grade glioma (HGG). Glioblastoma (GBM), a grade 4 glioma, constitutes 75% of all HGG cases and displays the poorest survival rates. The standard therapy for newly diagnosed glioblastoma (nGBM) is the STUPP protocol, introduced in 2002, which includes maximum safe resection, concurrent radiotherapy, and adjuvant temozolomide (TMZ) chemotherapy. The median overall survival (mOS) for GBM patients was 14.6 months, and the median progression-free survival (mPFS) was 6.9 months [2]. Since 2016, the mOS for nGBM patients has extended to 20.9 months with the addition of tumor-treating fields (TTFields) to the STUPP regimen [3]. However, the recurrence rate of HGG patients treated with standard therapy remains as high as 90%, with a 5-year survival rate below 10% [4]. Currently, there is no uniform treatment criteria for recurrent HGG (rHGG), and various options such as re-operation, re-irradiation, chemotherapy, TTFields, and targeted therapy are commonly used. However, there is insufficient evidence from randomized trials to demonstrate the efficacy of these treatments in prolonging the survival of rHGG patients [5]. Therefore, the exploration of novel targeted therapeutic drugs has become a prominent area of research, and the 2023 National Comprehensive Cancer Network (NCCN) guidelines for the management of rHGG recommend enrollment in clinical trials.

Anlotinib, a small molecule multi-target tyrosine kinase inhibitor (TKI), demonstrates potent inhibition against vascular endothelial growth factor receptor (VEGFR), platelet-derived growth factor receptor (PDGFR), fibroblast growth factor receptor (FGFR), and c-Kit, resulting in antitumor angiogenic effects and suppression of tumor growth [6]. Anlotinib has been approved by the China Food and Drug Administration (CFDA) for the treatment of non-small cell lung cancer (NSCLC) [7], small cell lung cancer [8], soft tissue sarcoma [9], and medullary thyroid carcinoma [10]. Furthermore, anlotinib is included in the guidelines of the Chinese Society of Clinical Oncology (CSCO) for the treatment of renal cancer [11] and esophageal cancer [12]. Numerous clinical studies have also demonstrated the efficacy of anlotinib in liver cancer [13], cervical cancer [14], and bone malignant tumors [15]. Importantly, anlotinib can penetrate the blood-brain barrier, and the ALTER0303 post-hoc analysis [16] is the first study to confirm its efficacy in controlling NSCLC brain metastases. Anlotinib’s extensive antitumor activity, including its effectiveness in the CNS, suggests it may have potential anti-glioma activity.

Glioma is a prototypical vascular-dependent tumor, with neovascularization being a prominent feature of GBM. VEGF, PDGF, and FGF play significant roles in promoting tumor vascularization, with the VEGF/VEGFR pathway being the predominant angiogenic signaling pathway in glioma [17]. The expression level of VEGF in HGG is notably higher compared to LGG [18]. Glioma microvascular endothelial cells exhibit a substantial level of VEGFR expression on their surface [17]. Consequently, antiangiogenic targeted therapies, such as monoclonal antibodies or multi-target tyrosine kinase inhibitors (TKIs), are increasingly used for GBM treatment. Bevacizumab (Bev) is an anti-VEGF IgG1 humanized monoclonal antibody, specifically targeting the VEGF/VEGFR signaling pathway. Although studies investigating the effects of Bev on patients with recurrent glioblastoma (rGBM) [19–21] and newly diagnosed GBM (nGBM) [22] did not demonstrate an enhancement in overall survival (OS), the US Food and Drug Administration (FDA) approved Bev in 2009 for treating rGBM based on improvements in progression-free survival (PFS). Another multi-target TKI, regorafenib, has been recommended for the treatment of rGBM in the 2020 NCCN guidelines. The REGOMA study [23] showed that regorafenib administration led to a statistically significant extension in median overall survival (mOS) compared to lomustine in rGBM (7.4 months vs. 5.6 months, hazard ratio [HR] 0.5, *P* = 0.0009).

Anlotinib, like regorafenib, is a novel multi-target TKI that has shown effectiveness in both basic and clinical studies for glioma. Anlotinib effectively inhibited the proliferation, migration, and invasion of human GBM cells (A172, U87, U251) in a dose-dependent manner through the mediation of the Janus kinase 2 (JAK2) / signal transducer and activator of transcription 3 (STAT3) signaling pathway [24]. Three recent retrospective studies [25–27] have demonstrated that anlotinib, as a monotherapy, in combination with chemotherapy, or in combination with radiotherapy, exhibits favorable therapeutic efficacy in the treatment of rHGG (median progression-free survival (mPFS): 4–6 months, median overall survival (mOS): 8–12 months). Given the approval of Bev for rGBM and the broad-spectrum antitumor activity of anlotinib, this study aims to evaluate the efficacy and safety of anlotinib alone or in combination with Bev for rHGG.

## Methods

### Study design and participants

This prospective single-arm, open-label Phase II trial was conducted in China (NCT04822805, 30/03/2021). Eligible participants had a histological diagnosis of HGG (WHO grade III/IV, according to the 2016 WHO glioma classification) with first or subsequent recurrences after surgery followed by radiotherapy and TMZ chemotherapy. The inclusion criteria were as follows: (1) Age ≥ 18 years old; (2) Eastern Cooperative Oncology Group (ECOG) performance status (PS) score ranging from 0 to 2; (3) Evaluable intracranial lesions defined as contrast-enhancing tumors with a minimum diameter of 10 mm on two axial slices; (4) If the participant received chemotherapy, their toxicity level must have returned to grade 1 or lower; (5) Adequate hematologic (i.e., white blood cells ≥ 3.0 × 10^9^/L, hemoglobin ≥ 10 g/dl, platelets ≥ 75 × 10^9^/L), coagulation, hepatic, renal, and cardiac functions; (6) Both men and women of gestational age must agree to use effective contraception throughout the study; (7) The participant must voluntarily agree to participate in the study and provide written informed consent.

Exclusion criteria were as follows: any factors affecting oral medications; uncontrolled high blood pressure and infection; clinically significant cardiovascular disease; patients with any bleeding, unhealed wounds, ulcers, fractures, or received invasive operations within the past 4 weeks; urine protein is ≥++; arterial/venous thrombosis within the last 6 months; a history of immunodeficiency and psychotropic drug; receiving re-resection or re-radiation without evaluable intracranial lesions; previous treatment with anti-angiogenesis targeted drugs (such as pazopanib, regorafenib, apatinib, etc.). The approval for this study was granted by the Clinical Trial Ethics Committee of Hangzhou Cancer Hospital, and all patients provided written informed consent before enrollment.

### Procedures

All patients received oral anlotinib 12 mg (weight ≥ 50 kg) or 10 mg (weight < 50 kg) on days 1–14, with each cycle repeated every 21 days until disease progression, unacceptable toxicity, death, or consent withdrawal. In cases where brain magnetic resonance imaging (MRI) examination revealed increased peritumoral edema severity compared to the previous cycle, and no clinically relevant worsening of symptoms was observed, patients received Bev 10 mg/kg intravenously for temporary treatment to alleviate edema. Adverse events (AEs) were documented and assessed using the National Cancer Institute Common Terminology Criteria for Adverse Events version 4.0 (NCI-CTCAE 4.0). For patients experiencing grade 3/4 treatment-related toxicities, dose reduction of anlotinib to 10 mg or 8 mg was considered.

Scheduled visits and brain MRI scans were conducted at week 3 and subsequently repeated every 6 weeks until there was evidence of disease progression. Additionally, physical and laboratory examinations (blood routine, biochemistry, coagulation function, thyroid function, and urine routine), electrocardiogram, cardiac ultrasound, abdominal B-ultrasound, and chest CT were recorded. Tumor response was evaluated by the investigators and radiologists based on the Response Assessment in Neuro-Oncology (RANO) criteria for high-grade gliomas.

Safety data documentation occurred during the course of treatment and within a month following treatment completion. Post-study data were also collected, and the study followed up on treatment survival status and collected information on subsequent therapies every 2 months.

### Outcomes

The primary endpoint of this study was investigator-assessed median progression-free survival (mPFS), defined as the time from the start of anlotinib alone or in combination with Bev administration to disease progression or death from any cause. Disease status was assessed by clinicians as complete response (CR), partial response (PR), stable disease (SD), or progressive disease (PD). Secondary endpoints included median overall survival (mOS), defined as the time from the start of anlotinib alone or in combination with Bev treatment until death from any cause; objective response rate (ORR), which represented the percentage of patients with a confirmed CR and PR; disease control rate (DCR), representing the percentage of patients with a confirmed CR, PR, and SD; and safety.

### Statistical analysis

As this is a phase II trial with mPFS as the main endpoint, historical outcomes in rHGG were considered for comparison. Reported mPFS for patients receiving TMZ [28], lomustine [20], or carboplatin [21] chemotherapy ranged from 1.5 to 3.8 months, while mPFS for patients receiving antiangiogenic therapy, including Bev [21], and multi-target TKIs (regorafenib, sunitinib, pazopanib, cediranib, sorafenib, and axitinib) [23, 29–35], ranged from 1.4 to 4.1 months. It was hypothesized that anlotinib ± Bev could prolong mPFS from 2.5 months to 5.0 months, with a one-sided test at a significance level of 0.05, a power of 80%, and a drop-out rate of less than 15%. Accordingly, a sample size of 27 participants was calculated using the PASS 11.0 software.

Kaplan–Meier method was employed to estimate mOS and mPFS, and the log-rank test was used to compare these outcomes between groups. HR for disease progression with a 95% CI was calculated using the Wald method. Fisher’s exact test was used to compare the proportions of patients achieving objective response and SD, as well as the incidence rate of AEs. Statistical analyses were conducted using IBM SPSS 22.0 software.

## Results

**Fig. 1:**
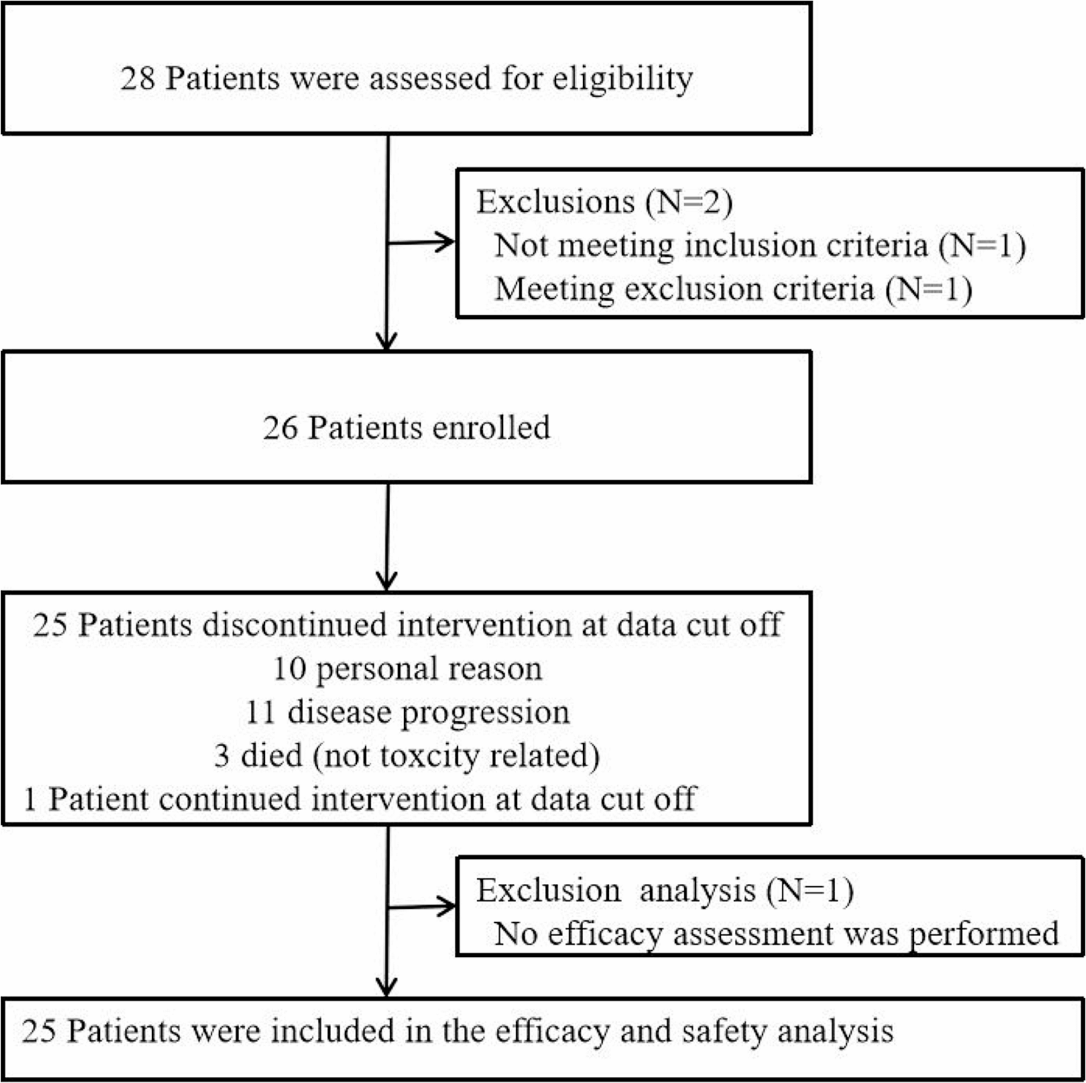
The study flow diagram

Between April 2020 and September 2022, a total of 28 patients were screened for eligibility, and 26 eligible patients with rHGG were enrolled in the study (Fig. 1). However, one patient was excluded from the efficacy and safety assessment due to voluntary withdrawal of consent and discontinuation of treatment prior to the initial assessment. Ultimately, 25 patients were included in the efficacy and safety analysis.

For the 25 patients included in the study, 18 patients received anlotinib alone, while 7 patients received a combination regimen with Bev. The demographic and baseline characteristics of the patients are summarized in Table 1. The study began in April 2020, the patients were enrolled according to the 2016 WHO glioma classification criteria. Based on this classification, 15 (83%) patients with glioma grade IV and 3 (17%) patients with glioma grade III were enrolled in the anlotinib group. 7 (100%) patients with glioma grade IV were enrolled in the anlotinib + Bev group. According to the 2021 WHO glioma classification, 14 (78%) patients in the anlotinib group and 7 (100%) patients in the anlotinib + Bev group were diagnosed with glioblastoma (IDH wildtype, grade 4). 2 (11%) patients with astrocytoma (IDH mutant, grade 4) and 2 (11%) patients with diffuse astrocytoma (NOS) were enrolled in the anlotinib group. In both groups, the majority of patients were male, had an Eastern Cooperative Oncology Group (ECOG) performance status score of 0–1 and presented with only one recurrent tumor. However, in the anlotinib group, there was a higher percentage of patients who had received 1–3 lines of therapy, undergone previous re-operation, and received re-radiation compared to the anlotinib + Bev group.

**Table 1:** Patient demographic and baseline characteristics

Characteristics	Anlotinib n = 18	Anlotinib + Bev n = 7
Age		
Median age, years (range)	52 (35–63)	59 (54–67)
≤ 53	11 (61%)	0
>53	7 (39%)	7 (100%)
Gender		
Male	13 (72%)	5 (71%)
Female	5 (28%)	2 (29%)
ECOG PS score		
0–1	14 (78%)	6 (86%)
2	4 (22%)	1 (14%)
Number of recurrent tumor		
1	12 (67%)	7 (100%)
2–3	6 (33%)	0
Pathology (2016 WHO glioma classification)		
Grade IV	15 (83%)	7 (100%)
Glioblastoma, IDH wildtype	13 (72%)	7 (100%)
Glioblastoma, IDH mutant	1 (5%)	0
Glioblastoma, NOS	1 (5%)	0
Grade III	3 (17%)	0
Anaplastic astrocytoma, IDH wildtype	2 (11%)	0
Anaplastic oligodendroglioma, IDH mutant	1 (5%)	0
Pathology (2021 WHO glioma classification)		
Glioblastoma, IDH wildtype, grade 4	14 (78%)	7 (100%)
Astrocytoma, IDHmutant, grade 4	2 (11%)	0
Diffuse astrocytoma, NOS	2 (11%)	0
Treatment before enrollment for recurrence		
without any intervention	6 (33%)	5 (72%)
1 line treatment	5 (28%)	2 (28%
3 line treatment	1 (5%)	0
re-radiation	1 (5%)	0
re-radiation + 1 line treatment	1 (5%)	0
re-radiation + 2 line treatment	1 (5%)	0
re-operation + 1 line treatment	1 (5%)	0
re-operation + re-radiation + 1 line treatment	1 (5%)	0
re-operation + re-radiation + 2 line treatment	1 (5%)	0

Abbreviation: IDH, isocitrate dehydrogenase

The initial dose of anlotinib was 12 mg for 21 patients and 10 mg for 4 patients. The median treatment cycle for the anlotinib group was 5 (range 1 to 22). In the anlotinib + Bev group, the median treatment cycle of anlotinib was 11 (range 3–28). Notably, the dose of Bev used in this study was relatively small, with 5 patients receiving two cycles of Bev and 2 patients receiving only one cycle of Bev. None of the patients received long-term glucocorticoid therapy. As of the data-cutoff date of 31 May 2023, the median follow-up was 9.9 months (interquartile range (IQR) 5.9–15.1). Out of the 25 patients, 24 had discontinued study treatment, and 21 patients had passed away. Post-study treatments were administered to 67% (12 patients) in the anlotinib group and 43% (3 patients) in the anlotinib + Bev group. These post-study therapies included chemotherapy, radiotherapy, surgery, targeted therapy, and TTFields.

**Fig. 2:**
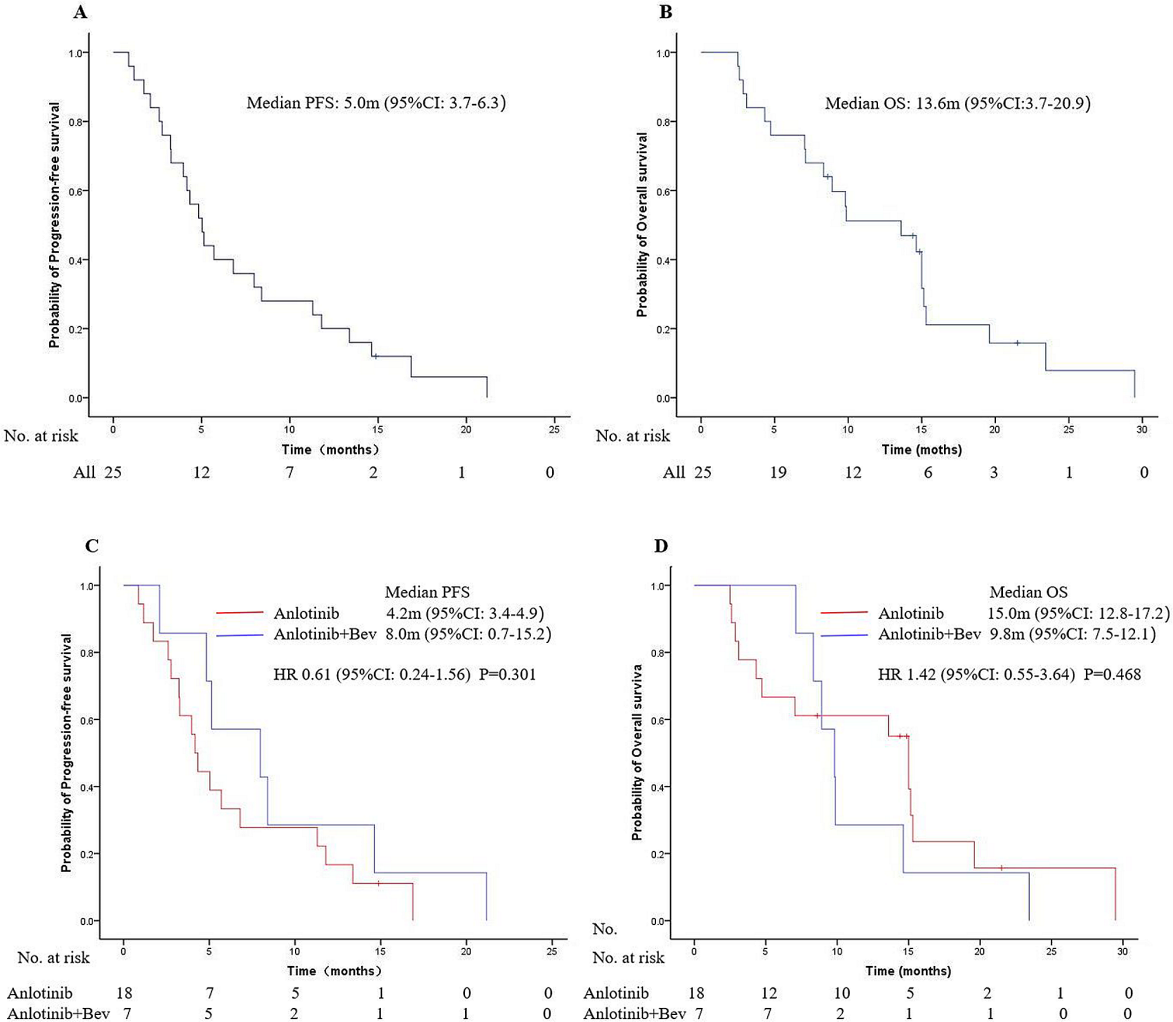
illustrates the Kaplan-Meier curves of progression-free survival (A) and overall survival (B) of all patients. Additionally, Fig. 2 also displays the Kaplan-Meier curves for progression-free survival (C) and overall survival (D) for the comparison between the anlotinib and anlotinib + bevacizumab groups

For all patients, the median PFS (mPFS) was 5.0 months (95% CI 3.7–6.3) (Fig. 2A), and the median OS (mOS) was 13.6 months (95% CI 3.7–20.9) (Fig. 2B). Specifically, patients treated with anlotinib alone had an mPFS of 4.2 months (95% CI 3.4–4.9) and an mOS of 15.0 months (95% CI 12.8–17.2). On the other hand, patients who received anlotinib + Bev treatment exhibited an mPFS of 8.0 months (95% CI 0.7–15.2) and an mOS of 9.8 months (95% CI 7.5–12.1). In the anlotinib + Bev group, three patients received two cycles of Bev close to the time of death, resulting in mPFS time being similar to mOS time. Nevertheless, there was no significant difference in mPFS (HR 0.61, 95% CI 0.24–1.56, *P* = 0.301, Fig. 2C) or mOS (HR 1.42, 95% CI 0.55–3.64, *P* = 0.468, Fig. 2D) between the anlotinib and anlotinib + Bev groups.

**Fig. 3:**
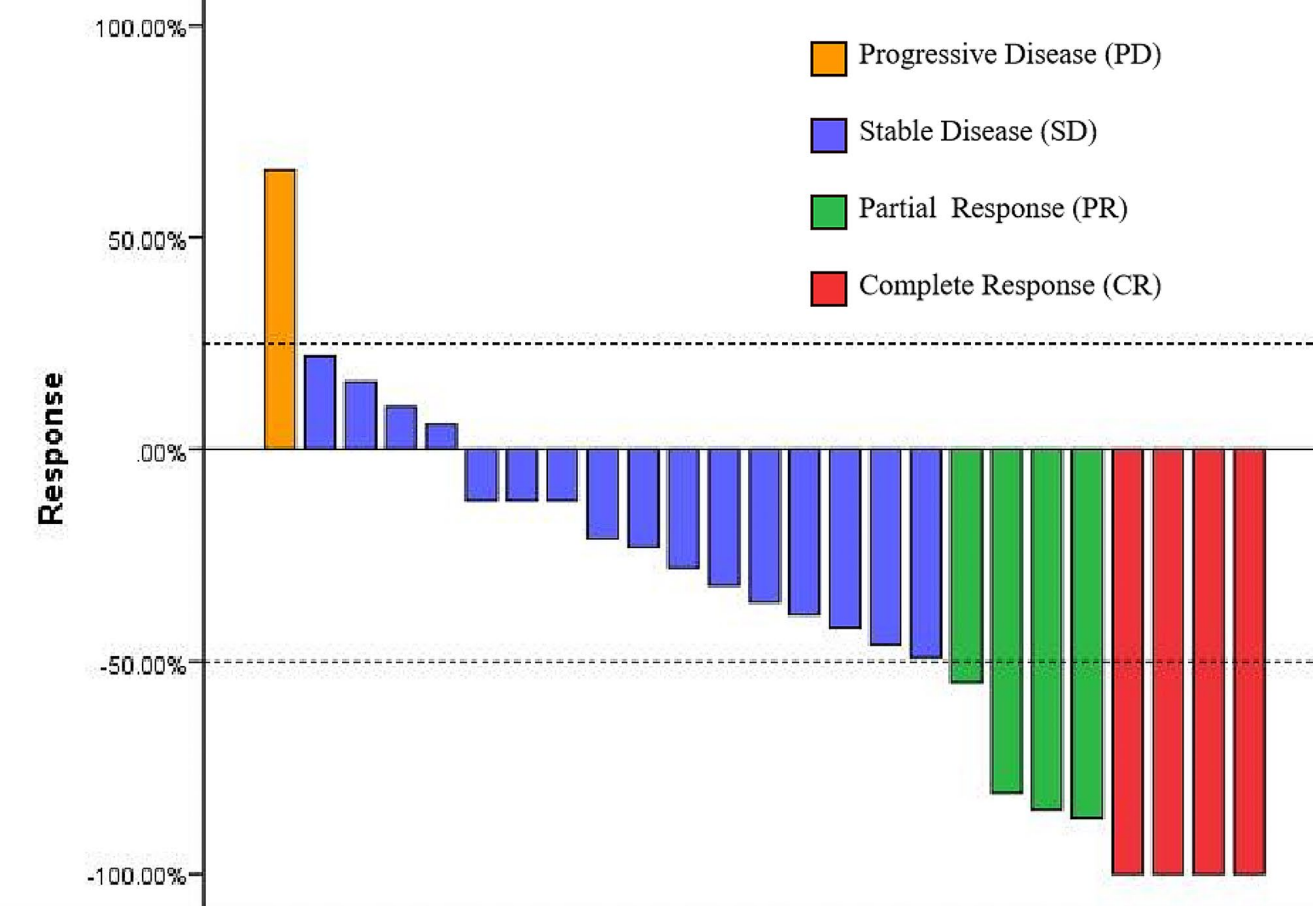
The best response to treatment

Figure 3 presents the best response to treatment. Among all patients, 4 (16%) achieved complete response (CR) and 4 (16%) achieved partial response (PR), resulting in an objective response rate (ORR) of 32%. Moreover, 16 (64%) patients exhibited stable disease (SD), while only 1 (4%) patient showed confirmed progressive disease (PD). The disease control rate (DCR), which includes CR, PR, and SD, was 96% (Table 2).

**Table 2:** Survival and response endpoints for all patients

	All n = 25	Anlotinib n = 18	Anlotinib + Bev n = 7
**Median OS (95% CI)**	13.6 m (3.7–20.9)	15.0 m (12.8–17.2)	9.8 m (7.5–12.1)
**Median PFS (95% CI)**	5.0 m (3.7–6.3)	4.2 m (3.4–4.9)	8.0 m (0.7–15.2)
**Responses according to RANO criteria**			
Disease control	24 (96%)	17 (94%)	7 (100%)
Objective response	8 (32%)	5 (28%)	3 (43%)
Complete response	4 (16%)	3 (17%)	1 (14%)
Partial response	4 (16%)	2 (11%)	2 (29%)
Stable disease	16 (64%)	12 (66%)	4 (57%)
Progressive disease	1 (4%)	1 (6%)	0 (0)

The ORR and DCR for patients treated with anlotinib alone were 28% and 94%, respectively. In contrast, the ORR and DCR for patients who received anlotinib + Bev treatment were 43% and 100%, respectively (Table 2). The ORR of anlotinib + Bev group is higher than that of anlotinib group (43% vs. 28%, *p* = 0.02), which may be due to the limited size of the sample. However, there was no significant difference in the rate of achieving SD (57% vs. 66%, *p* = 0.052). It should be noted that due to the small number of enrolled patients, univariate and multivariate analyses of mPFS and mOS could not be performed to evaluate the impact of different clinical features.

Our research encompassed various grades of HGG, with particular emphasis on GBM due to its significantly poorer prognosis, necessitating separate analysis. 21 (84%) patients were diagnosed with GBM (IDH wildtype, grade 4, according to the 2021 WHO glioma classification). Table 3 shows the survival and response endpoints for rGBM (IDH wildtype, grade 4). For these 21 patients, the mPFS was 5.0 months (95% CI 3.8–6.2), and the mOS was 13.6 months (95% CI 7.0-20.2). In the anlotinib group, patients had an mPFS of 4.2 months (95% CI 3.5–4.8) and an mOS of 15.0 months (95% CI 13.2–16.8). In the anlotinib + Bev group, patients exhibited an mPFS of 8.0 months (95% CI 0.7–15.2) and an mOS of 9.8 months (95% CI 7.5–12.1). There was no significant difference in mPFS (HR 0.57, 95% CI 0.21–1.5, *P* = 0.259) or mOS (HR 1.47, 95% CI 0.55–3.95, *P* = 0.446) between the anlotinib and anlotinib + Bev groups. The ORR and DCR for 21 patients were 33% and 95%, respectively. There were no significant differences in the rates of achieving objective response (29% vs. 43%, *p* = 0.187) or stable disease (64% vs. 57%, *p* = 0.301) between the anlotinib and anlotinib + Bev groups. These survival and response data of rGBM are similar to rHGG.

**Table 3:** Survival and response endpoints for Glioblastoma (IDH wildtype, grade 4)

	Glioblastoma n = 21	Anlotinib n = 14	Anlotinib + Bev n = 7
**Median OS (95% CI)**	13.6 m (7.0−20.2)	15.0 m (13.2–16.8)	9.8 m (7.5–12.1)
**Median PFS (95% CI)**	5.0 m (3.8–6.2)	4.2 m (3.5–4.8)	8.0 m (0.7–15.2)
**Responses according to RANO criteria**			
Disease control	20 (95%)	13 (93%)	7 (100%)
Objective response	7 (33%)	4 (29%)	3 (43%)
Complete response	3 (14%)	2 (14%)	1 (14%)
Partial response	4 (19%)	2 (14%)	2 (29%)
Stable disease	13 (62%)	9 (64%)	4 (57%)
Progressive disease	1 (5%)	1 (7%)	0

Overall, the incidence of treatment-related adverse events (AEs) of any grade in the anlotinib + Bev group was higher than that in the anlotinib group (100% vs. 78%, *p* = 0.041) (Table 4). Most of the treatment-related AEs were of grade 1 or 2. The most common grade 1 or 2 AEs in the anlotinib + Bev and anlotinib groups were thrombocytopenia (71% vs. 39%), leukopenia (57% vs. 22%), proteinuria (29% vs. 22%), and fatigue (43% vs. 17%). The most common grade 3 AEs in the anlotinib + Bev and anlotinib groups were hypertension (14% vs. 11%) and neutropenia (14% vs. 6%). There were no grade 4 AEs reported, and no treatment-related deaths occurred. Treatment-related bleeding was rare, with only one case (14%) of grade 2 epistaxis observed in the anlotinib + Bev group.

**Table 4:** Treatment-related adverse events

	Anlotinib n = 18			Anlotinib + Bev n = 7		
	Any grade	Grade 1–2	Grade 3	Any grade	Grade 1–2	Grade 3
Any adverse event	14 (78%)	13 (73%)	5 (28%)	7 (100%)	7 (100%)	3 (43%)
Thrombocytopenia	8 (44%)	7 (39%)	2 (11%)	5 (71%)	5 (71%)	0
Leukopenia	4 (22%)	4 (22%)	0	4 (57%)	4 (57%)	1 (14%)
Proteinuria	4 (22%)	4 (22%)	0	2 (29%)	2 (29%)	0
Fatigue	3 (17%)	3 (17%)	0	3 (43%)	3 (43%)	0
Neutropenia	3 (17%)	2 (11%)	1 (6%)	2 (29%)	1 (14%)	1 (14%)
Hypertension	4 (22%)	2 (11%)	2 (11%)	1 (14%)	0	1 (14%)
Hand-foot syndrome	2 (11%)	2 (11%)	0	1 (14%)	1 (14%)	0
Oropharyngeal pain	2 (11%)	2 (11%)	0	2 (29%)	2 (29%)	0
Oral ulceration	1 (6%)	1 (6%)	0	1 (14%)	1 (14%)	0
Oral infection	1 (6%)	1 (6%)	0	1 (14%)	1 (14%)	0
Dysphonia	0	0	0	1 (14%)	1 (14%)	0
Anemia	0	0	0	1 (14%)	1 (14%)	0
Hypothyroidism	1 (6%)	1 (6%)	0	0	0	0
ALT elevation	0	0	0	1 (14%)	1 (14%)	0
AST elevation	0	0	0	1 (14%)	1 (14%)	0
γ-GT elevation	0	0	0	1 (14%)	1 (14%)	0
Epistaxis	0	0	0	1 (14%)	1 (14%)	0

Abbreviations: Bev, bevacizumab; ALT, Alanine aminotransferase; AST, Asparte aminotransferase; γ-GT, Gamma-glutamyltransferase

Dose reduction due to treatment-related AEs was performed in 6 patients. In the anlotinib group, 3 (17%) patients had their dose reduced to 10 mg/day, and 1 patient (6%) had their dose reduced to 8 mg/day. In the anlotinib + Bev group, 2 (29%) patients had their dose reduced to 10 mg/day. No patient discontinued treatment due to AEs.

## Discussion

This is a prospective study aimed at evaluating the efficacy of anlotinib alone or in combination with Bev in patients with HGG experiencing their first or subsequent recurrences. For all patients, the mFS was 5.0 months (95%CI 3.7–6.3) and mOS was 13.6 months (95%CI 3.7–20.9), respectively. The ORR was 32%, and the DCR was 96%. It is pointed out that the survival and response data of rGBM exhibit similarities to those of rHGG. For rGBM patients, there were no significant differences observed in mPFS, mOS, ORR, or DCR between the groups receiving anlotinib alone and those receiving anlotinib in combination with Bev. However, the incidence of treatment-related AEs of any grade was higher in the anlotinib + Bev group (100%) compared to the anlotinib group (78%), and this difference was statistically significant (*p* = 0.041). When compared to historical data, our research demonstrated improved survival outcomes in terms of mPFS and mOS. The NCCN guidelines recommend chemotherapy (Temozolomide [TMZ], Lomustine, and Carboplatin) and antiangiogenic therapy (Bevacizumab and Regorafenib) as therapeutic agents for recurrent HGG (rHGG). Historical outcomes from previous studies in rHGG patients showed mPFS ranging from 1.5 to 3.8 months for those who received TMZ [28], Lomustine [20], or Carboplatin [21] chemotherapy, and mPFS of 2.0 to 4.2 months for those who received antiangiogenic therapy with Bevacizumab [19] or Regorafenib [23]. Additionally, the reported mOS was 7.5 to 8.6 months for patients treated with chemotherapy [21, 22, 28] and 7.4 to 9.2 months for those who received antiangiogenic therapy [19, 23]. In contrast, our study’s results exceeded the data reported in the aforementioned studies.

Bev is a humanized monoclonal antibody that targets VEGF and is the first and most extensively used antiangiogenic therapy approved for rHGG. It is important to note that there is a discrepancy in the dosage of Bev used for treatment between this study and other relevant researches. In this trial, a temporary treatment of intravenous Bev at 10 mg/kg was administered to reduce peritumoral edema. However, in other studies [19, 21], Bev at 10 mg/kg was administered intravenously every 2 weeks as part of conventional anti-tumor therapy. The dosage of Bev administered in our research was notably lower than that used in previous studies. Despite the addition of Bev to anlotinib, this study did not demonstrate a survival advantage, including in terms of mPFS or mOS. Conversely, other studies have shown that adding Bev to chemotherapy significantly extended the mPFS time [19, 21].

Another class of antiangiogenic targeted therapy is multi-target TKIs. Among them, regorafenib is the only multi-target TKI recommended for rGBM in the NCCN guideline. Regorafenib can suppress the function of angiogenic (VEGFR, TIE2), stromal (PDGFR, FGFR), and oncogenic (KIT, RET, RAF-1, BRAF) receptor tyrosine kinases [6]. The efficacy of combining regorafenib with lomustine was evaluated in the REGOMA study for rGBM. The combined regimen prolonged the mOS from 5.6 to 7.4 months (HR = 0.5, *P* = 0.0009) and slightly improved the mPFS from 1.9 months to 2.0 months (HR = 0.65, *P* = 0.022) [23]. Anlotinib, similar to regorafenib, is a novel multi-target TKI that demonstrates significant antineoplastic effects on VEGFR, with comparatively lower effects on c-kit, PDGFR, and FGFR [6]. Anlotinib effectively suppresses tumor angiogenesis and tumor cell proliferation by blocking PI3K-AKT, MAPK/ERK, and RAF/MRK signaling pathways [6]. Recent retrospective studies have demonstrated that anlotinib alone [25], combined with TMZ [26], or combined with radiotherapy [27] has shown good therapeutic effects on rHGG (mPFS: 4–6 months, mOS: 8–12 months). Additionally, Chen et al. [36] reported a prospective, phase II clinical trial of anlotinib combined with the STUPP regimen for newly diagnosed glioblastoma (nGBM) (NCT04119674). The results showed a mPFS of 10.9 months and a mOS of 18.7 months, which were superior to the reported historical data of the STUPP regimen (mPFS: 6.9 months, mOS: 14.6 months). However, other multi-target TKIs failed to demonstrate definitive therapeutic benefits for rHGG. Pan et al. showed that the best efficacy evaluation of sunitinib for rHGG was stable disease (SD), and no partial response (PR) or complete response (CR) occurred [30]. Hutterer et al. also reported that there was no objective remission for rGBM treated with sunitinib, and high-dose administration led to persistent toxic reactions [32]. Similarly, pazopanib [32], cediranib [29], sorafenib [34], and axitinib [35] were found to be ineffective in improving outcomes for patients with rGBM.

Anlotinib offers certain advantages over other multi-target TKIs in terms of its mechanism of action. It targets more receptors than sorafenib, sunitinib, and pazopanib [6]. Anlotinib has exhibited an 20-fold stronger inhibitory impact on VEGF2/3 compared to sunitinib [37]. In a xenograft animal model of U87-MG, the inhibition rate of GBM with 6 mg/kg of anlotinib was 88%. Xu et al. [24] evaluated the molecular mechanisms of anlotinib in GBM and found that it significantly restricted the proliferation, migration, and invasion of human GBM cells (A172, U87, U251) in a dose-dependent manner through the mediation of the JAK2/STAT3/VEGFA signaling pathway. Moreover, the angiogenic activity of human umbilical vein endothelial cells was suppressed by tumor supernatant obtained from GBM cells treated with anlotinib.

In terms of safety, the combination of anlotinib with Bevacizumab in this trial demonstrated an expected safety profile, with no unexpected adverse events (AEs) or new safety signals identified. The primary grade 1 or 2 AEs in the anlotinib + Bev group and anlotinib-alone group were thrombocytopenia (71% vs. 39%). Patients treated with regorafenib in the REGOMA study [23] reported a lower rate of grade 1/2 (20%) thrombocytopenia than those receiving anlotinib alone in our present study. The incidence rates of grade 1/2 thrombocytopenia for the anlotinib group (39%) in our study were comparable to the rates reported in the trial of anlotinib for advanced hepatocellular carcinoma (36%) [13]. However, it’s important to note that the addition of Bev can lead to aggravated thrombocytopenia, as evident by the occurrence of grade 1/2 thrombocytopenia in 71% of patients in the anlotinib + Bev group. The incidence of all-grade thrombocytopenia was reported to be 23% and 55%, respectively, in the treatment of Bev alone or Bev + carboplatin for rGBM [21]. In our study, 2 (11%) out of 18 patients in the anlotinib group experienced grade 3 thrombocytopenia. A careful evaluation of blood routine for early onset of AEs might enable better tolerability. The rates of grade 3 hypertension and neutropenia observed in this study were found to be similar to those reported in previous clinical trials investigating the use of Bev in rGBM [19, 21]. Regarding bleeding AEs, it is a significant concern in the use of antiangiogenic therapy, especially in individuals with CNS tumors. The incidence of CNS hemorrhage and other bleeding was reported to be 5% and 26%, respectively, in the treatment of Bev alone for rGBM [21]. In our study, the incidence of hemorrhage was 14%, mainly consisting of epistaxis without CNS hemorrhage. This difference was attributed to significantly lower exposure to Bev in this trial, and the enrolled patient cohort was small.

However, this research has some limitations. Firstly, the study has a relatively limited sample size and is a single-center design. Secondly, the inclusion of patients diagnosed with grade III and IV glioma and irregular use of Bev in a small number of patients complicated the interpretation of the results.

## Conclusions

In conclusion, the results of this study demonstrate that anlotinib, either alone or in combination with Bev, shows promising efficacy and a favorable safety profile in the treatment of rHGG. Anlotinib emerges as a potent and active therapeutic option for glioma management. Considering these encouraging findings, conducting a randomized phase 2 trial in recurrent glioblastoma of anlotinib vs. lomustine is being planned.

## Data Availability

All data generated or analyzed during this study are included in this published article.

## Abbreviations

Bev: Bevacizumab
mPFS: Median progression-free survival
mOS: Median overall survival
ORR: Objective response rate
DCR: Disease control rate
CNS: Central nervous system
WHO: World Health Organization
LGG: Low-grade glioma
HGG: High-grade glioma
GBM: Glioblastoma
nGBM: Newly diagnosed glioblastoma
TMZ: Temozolomide
TTFields: Tumor-treating fields
rHGG: Recurrent HGG
NCCN: National Comprehensive Cancer Network
VEGFR: Vascular endothelial growth factor receptor
TKI: Tyrosine kinase inhibitor
PDGFR: Platelet-derived growth factor receptor
FGFR: Fibroblast growth factor receptor
CFDA: China Food and Drug Administration
NSCLC: Non-small cell lung cancer
CSCO: Chinese Society of Clinical Oncology
TKIs: Tyrosine kinase inhibitors
rGBM: Recurrent glioblastoma
OS: Overall survival
FDA: Food and Drug Administration
PFS: Progression-free survival
mOS: Median overall survival
HR: Hazard ratio
JAK2: Janus kinase 2
STAT3: Signal transducer and activator of transcription 3
ECOG: Eastern Cooperative Oncology Group
PS: Performance status
MRI: Magnetic resonance imaging
AEs: Adverse events
NCI-CTCAE 4.0: National Cancer Institute Common Terminology Criteria for Adverse Events version 4.0
RANO: Response Assessment in Neuro-Oncology
CR: Complete response
SD: Stable disease
PD: Progressive disease
ORR: Objective response rate
DCR: Disease control rate
OS: Overall survival
